# Exploring stakeholders’ experiences in co-creation initiatives for clinical nursing education: a qualitative study

**DOI:** 10.1186/s12912-023-01582-5

**Published:** 2023-11-06

**Authors:** Kristin Laugaland, Ingunn Aase, Monika Ravik, Marianne Thorsen Gonzalez, Kristin Akerjordet

**Affiliations:** 1 SHARE—Centre for Resilience in Healthcare, Faculty of Health Sciences, University of Stavanger, Kjell Arholms Gate, Stavanger, 4036 Norway; 2 Faculty of Health and Social Sciences, University of South-Eastern Norway—Campus Porsgrunn, Porsgrunn, Norway

**Keywords:** Co-creation, Nursing education, Clinical nursing education, Workshops, Experiences

## Abstract

**Background:**

Co-creation is an emerging approach in nursing education, wherein academics engage in multi-stakeholder collaborations to generate knowledge, ideate solutions, promote sustainability, and enhance educational quality. However, knowledge on stakeholders’ experiences in participation in co-creation initiatives for nursing education is scarce. This study aimed to explore the experiences of student nurses, nurse educators, and e-learning designers in co-creation initiatives to design and develop a digital educational resource for clinical nursing education.

**Methods:**

The study adopted an exploratory qualitative design. Data were collected via three separate semi-structured focus group interviews with student nurses (*n* = 7), nurse educators (*n* = 8), and e-learning designers (*n* = 3) who participated in co-creation workshops. Collected data were then thematically analyzed.

**Results:**

Three themes related to the participants’ experiences emerged: (1) The co-creation workshops were enjoyable, useful, and instructive; (2) power imbalances influenced the students’ engagement; and (3) contextual factors influenced the participants’ overall engagement.

**Conclusions:**

This study shows that co-creation through workshops is a novel, enjoyable, and instructive approach that facilitates knowledge exchange. It also highlights the needs and experiences of stakeholders, especially student nurses. However, the use of co-creation in nursing education presents some challenges. Recognizing and managing power differentials are essential for successful co-creation in clinical nursing education, alongside a mindset of collaboration and mutuality. Future research is required to systematize knowledge about the benefits and impacts of the processes and outcomes of co-creation initiatives, including stakeholders’ motivation, barriers, and facilitators to participation in co-creation, to improve the quality of clinical nursing education.

**Supplementary Information:**

The online version contains supplementary material available at 10.1186/s12912-023-01582-5.

## Background

Co-creation is a n ovel approach in educational practice and research, which has been gaining more attention in recent years [1]. Rethinking clinical education through co-creation initiatives to design new pedagogical methods that better prepare student nurses for current nursing practice is an urgent, ongoing need in nursing education [2]. In this regard, co-creation can encourage exchange and interaction among key stakeholders, which can consequently prompt improved practice and innovation [3]. Nevertheless, various overlapping terminologies of co-creation can be found in and across the education literature. These terminologies include co-design and co-production, wherein nuances in the meaning and application of these concepts depend on the area where they are applied [4]. However, the commonalities of these terms are that they are used more recently to describe specific participatory research actions. In this study, we appraise co-creation as an overarching construct that includes co-design and co-production approaches for guiding initiatives, as Vargas et al. [5] suggested.

Co-creation can be defined as the collaborative generation of knowledge by academics working alongside other key stakeholders (e.g., student nurses, educators, clinical practitioners, and designers) at all stages of an initiative, from problem identification to solution generation [6, 7]. Co-creation is considered to increase impact because engaging and empowering end users increase the probability of innovations being compatible with their needs, values, contexts, and norms, improving the chances of successful clinical implementation [8].

Participation in co-creation initiatives is important in improving the quality of clinical nursing education [9], as educational stakeholders’ unique expectations and perceptions are crucial in creating powerful learning environments and educational resources [10]. Hence, a co-creation initiative featuring the perceptions of end users is deemed essential in developing a digital educational resource compatible with specific needs and contexts of clinical education and ensuring a student-centered design [11–13]. Several beneficial outcomes of co-creation initiatives for nursing education have been documented, such as acquisition of knowledge, improved meta-cognitive skills, confidence, and awareness among students and enhancement of teacher–learner relationships and the quality of the educational design [1, 2].

However, few studies have examined co-creation initiatives in clinical settings [12], even though clinical education is a major part of undergraduate nursing programs. Nurse educators engaged in co-creation are usually confined to activities at the classroom level [14]. The approach is therefore often limited to learning and teaching methods, indicating that it does not permeate curriculum development in any meaningful sense [14, 15]. Engaging and empowering student nurses in different aspects of their education are essential, especially within clinical education and nursing home placements. Such approach will foster enriched learning and placement experiences that may increase student nurses’ interest in the care of older people [9, 15].

The novelty of co-creation initiatives in clinical nursing education implies the lack of systematic knowledge on stakeholders’ experiences in participation in such initiatives. As part of a larger study [9], this study implemented co-creation workshops among student nurses, nurse educators, registered nurse (RN) mentors, and e-learning designers. Workshops are often applied as strategies for stakeholder engagement in co-creation initiatives. However, a previous systematic review on co-production, including co-design and co-creation for nursing education, indicated that workshops were less frequently applied in nursing education than other activities such as individual interviews and focus group interviews, indicating the need for a more active engagement of key stakeholders in educational improvement processes [16]. This study aimed to explore how participation in co-creation workshops was experienced by student nurses, nurse educators, and e-learning designers. RN mentors’ experiences are reported elsewhere [17]. Acquiring an in-depth understanding of key stakeholders’ experiences is essential in building a stronger evidence base of the processes and outcomes of co-creation initiatives for nursing education to improve educational quality (e.g., [16, 18, 19]).

## Methods

### Design and setting

This study adopted an exploratory qualitative design [20]. Data were collected via semi-structured focus group interviews that aimed to explore participants’ experiences with co-creation workshops. Focus group interviews facilitate group interactions and discussions and were therefore expected to yield rich and in-depth data on the various experiences and opinions of participants in this study [21]. This study was conducted at a higher educational nursing institution in Norway in December 2019. The Standards for Reporting Qualitative Research were followed [22].

### Co-creation workshops

**Fig. 1:**
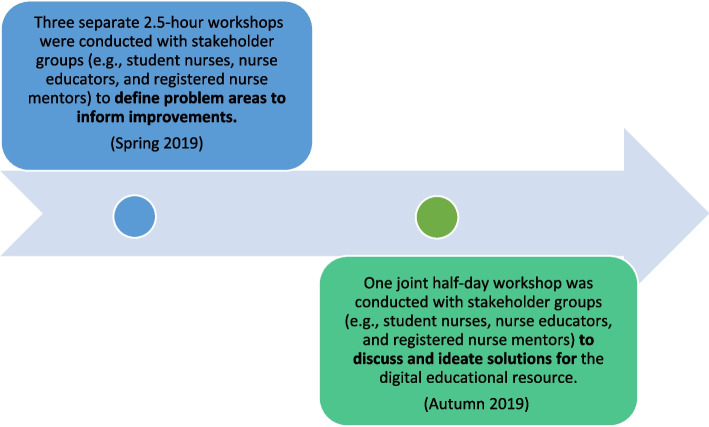
Co-creation initiatives

The co-creation initiative included three homogeneous workshops and one heterogeneous workshop, wherein participants used interactive exercises to share experiences, define problem areas, and ideate solutions (Fig. 1). The separate workshops aimed to explore stakeholders’ challenges with clinical placements in nursing homes; identify areas in need of improvement; and create informational, contextual, and educational content to be included in a digital educational resource. The workshops with nurse educators and student nurses were held at a location near the campus of the higher educational nursing institution, and each workshop lasted 2.5 h. The e-learning designers responsible for designing the digital educational resource also participated in the separate workshops. A joint workshop with RN mentors, student nurses, nurse educators, and e-learning designers was conducted to ideate solutions and provide input into the content and functionality of the resource, including how to accommodate key challenges. The joint workshop lasted 3.5 h. It started with lunch and a presentation about the workshop objectives and desired outcomes, followed by a summary of identified challenges. Participants worked in smaller heterogeneous groups of five to eight people. The groups were invited to reflect and discuss resources to be included in the digital educational resource. The workshop ended with a plenary session in which the various groups presented their thoughts and ideas to the whole group, and the facilitator summarized key discussions. Laugaland et al. [23] have previously detailed the description of the overall co-creation process. The outcomes of the co-creation workshops informed the design, content, and functionalities of the digital educational resource.

### Sample

The target group in this study comprised student nurses, nurse educators, and e-learning designers who participated in the co-creation workshops. The purposive criterion-based sampling strategy described in detail by Laugaland et al. [23] was used in the recruitment of participants. Student nurses with experience of clinical placements in nursing homes and nurse educators with experience overseeing first-year students receiving clinical education in nursing homes were included. These groups were considered to have the ability to share experience-based knowledge (e.g., [24]). Information meetings were held for second-year student nurses and relevant nurse educators with an open request to participate. A follow-up email was sent to eligible nurse educators with an invitation to participate in the study. Nurse educators and student nurses did not receive any compensation for their participation, which relied on their intrinsic motivation and willingness to participate. The e-learning designers responsible for designing the digital educational resource were recruited from the e-learning development department at the relevant higher educational nursing institution. They were approached by the research team with an open request to participate.

A total of 18 participants, including seven student nurses, eight nurse educators, and three e-learning designers consented to participate in the focus group interviews. The enrolled student nurses comprised five women and two men aged 20–29 years. All student nurses were second-year bachelor students at the time of enrollment, and half were student representatives in their class of study. All nurse educators were women aged 38–66 years. Their experience overseeing students in clinical placements in nursing homes ranged from 2 to 23 years. Participation frequency and continuity varied to some extent between the separate and joint workshops. Four of the interview participants did not attend the separate workshop, but most of the interview participants joined both separate and joint workshops. The participant characteristics are shown in Table 1.

**Table 1:** Characteristics of the participants

Stakeholder group	Age	Previous work experience	Sex
FGS: student nurses (*n* = 7)	20–29 years	1–4 years as health workers	5 women, 2 men
FGE: nurse educators (*n* = 8)	38–66 years	2–23 years as nurse educators	8 women
FGD: e-learning designers (*n* = 3)	33–44 years	2–10 years as e-learning designers	1 woman, 2 men

*FGS* Focus group students, *FGE *Focus group educators, *FGD* focus group e-learning designers

### Data collection

Semi-structured focus group interviews were held with the various stakeholder groups (student nurses, nurse educators, and e-learning designers) following the joint co-creation workshop in December 2019. A semi-structured interview guide comprising questions about the participants’ experiences in the co-creation workshops was used (Additional file 1). The separate focus group interviews allowed an exploration of how participation in the co-creation workshops was experienced differently by the various stakeholders. The focus group interviews were conducted by two experienced qualitative nurse academic researchers who participated in the co-creation workshops. The interviews were conducted face-to-face, recorded using a digital recorder, and transcribed verbatim. The interviews lasted approximately 40–50 min.

### Data analysis

An inductive thematic analysis based on the study by Braun and Clarke [25] guided the analysis of the transcribed text. The analysis was conducted in six phases: (1) becoming familiar with the data, (2) generating initial codes, (3) searching for themes, (4) reviewing themes, (5) defining and naming themes, and (6) producing the report. Three of the authors (KL, IA, MR), one of whom did not participate in the interviews, independently read the transcribed text to obtain a general impression and familiarize themselves with the transcripts. Preliminary ideas from across the transcripts were noted and discussed. To further identify patterns in the data, the first, second, and third authors (KL, IA, MR) manually coded the transcripts, highlighting relevant meaning units in line with the research questions. Finally, the coded data were grouped together and sorted into potential recurring themes covering the stakeholders’ experiences in participation in the co-creation workshops. All authors discussed and reached a consensus on the analysis by reviewing, modifying, and making final refinements to the themes and potential sub-themes.

### Ethical considerations

The study was approved by the Norwegian Centre for Research Data (reference number: 489,776). Participation was based on informed, voluntary, written consent, and the stakeholders were informed about their right to withdraw from the study at any point. Numbered identifiers were randomly assigned to each of the participants and their focus group designation (e.g., P1, FGS for participant 1, focus group students) to ensure confidentiality.

## Results

The analyses identified three themes with associated sub-themes (Table 2) relating to the stakeholders’ experiences in participating in the co-creation activities: (1) The co-creation workshops were enjoyable, useful, and instructive; (2) power imbalances influenced the students’ engagement; and (3) contextual factors influenced the participants’ overall engagement. These themes are described in more detail in the next few sections.

**Table 2:** Overview of the themes and associated sub-themes

Main themes	Sub-themes
The co-creation workshops were enjoyable, useful, and instructive.	• Sharing experiences and knowledge • Providing insight and gaining extended understanding of others’ perspectives • Interesting and exciting to participate • Students experience having a valuable voice
Power imbalances influenced the students’ engagement.	• A form of hierarchy • Internal tension and power imbalance • Nurse educators dominant in group discussions
Contextual factors influenced the participants’ overall engagement.	• Organization and structure of the workshop • Group composition and size • Stakeholders’ digital competence and attitudes

### The co-creation workshops were enjoyable, useful, and instructive

All stakeholder groups consistently commented that it was enjoyable, useful, and instructive to participate in the co-creation workshops and especially good to have the opportunity to discuss and share their experiences and knowledge. The stakeholders expressed that it was interesting and exciting to participate in the workshops and explained that they obtained an expanded understanding of others’ perspectives. They also found it useful to gain insights into how others think and reflect during the joint workshops. Several students emphasized that participation in the workshops made them feel that their voice was valued.

*“I think it was very enjoyable to take part in the workshops. It was a great initiative that helps our voices to be heard. That we [students] get to say what we believe is important.” (P1, FGS)*.

*“To learn about the challenges experienced by the nurse educator and registered nurse mentors in relation to clinical placement studies in nursing homes contributes to an increased understanding. That was useful and instructive.” (P4, FGS)*.

None of the stakeholder groups had previously participated in any co-creation workshops for developing digital educational resources. The stakeholders were impressed by the novelty and thoroughness of the process. The e-learning designers particularly underlined the value of participating alongside key stakeholders, especially students, when designing educational resources. They felt that gaining insights into the students’ challenges and problem areas was essential when developing educational resources.

*“Previously, we have not been very good at involving and engaging with students, who are our end users when designing and developing educational resources. My experience of participating here [in the workshops] is that it has been very useful. The students have valuable inputs and comments that we need to take into consideration.” (P2, FGD)*.

*“This process with extensive user involvement is very useful. I have never participated in such a thorough process before. We have learned a lot by participating. The students are our end users, so it has been very instructive participating in these workshops.” (P3, FGD)*.

The nurse educators also said that it was interesting and exciting to participate in the co-creation workshops and gain insights into different perspectives. Some nurse educators valued the voice of the students, and others emphasized the importance of engaging and learning more about the clinical perspectives experienced by nurse mentors.

*“I think it is so important to hear the students’ perspective, to hear what they think and what they are worried about concerning clinical placements.” (P1, FGE)*.

*“I think it was especially enjoyable engaging with registered nurse mentors from the clinical practice field to learn more about their view on things. They are the ones that we have the least dialogue with.” (P2, FGE)*.

Based on their participation in the joint co-creation workshops, a few nurse educators described gaining inspiration, new ideas, and insights into digital educational resources.

*“I think it was very nice to participate in the workshop, nice to collaborate with the students and registered nurse mentors. It was particularly interesting when we worked in smaller groups when students and mentors said something about what they felt we as nurse educators needed in terms of information. They [students and mentors] came up with good suggestions that I hadn’t thought of, but which I completely agree with.” (P3, FGE)*.

*“Today, I have learned more about digital resources that can be applied to clinical education. That has been exciting.” (P4, FGE)*.

### Power imbalances influenced the students’ engagement

The students saw power imbalances as an influential barrier to their engagement in the joint workshops. Concerns about power imbalances were raised by the e-learning designers and experienced by the students. Several students experienced a form of hierarchy during the joint workshop that negatively influenced their engagement and contribution. Some students said that it was easier to be more honest, open, and critical about current educational practices and problem areas that they had experienced in relation to clinical education in nursing homes during the separate workshop, wherein they were not affected by internal tension or power imbalances.

*“It was difficult to be critical of the nurse educators when we were in mixed groups together. I did not want to burden them, and I felt that I could not be completely honest when the nurse educators were present and did not agree with what we [students] said.” (P3, FGS)*.

Several students stated that the separate workshop was important during the problem identification phase because it allowed them to speak more freely and honestly.

*“I am very glad that the first workshop was only for students. It was easier to tell the truth and be more honest in a more critical way than today when we were all together [in the joint workshop].” (P2, FGS)*.

Some students also commented that it was more difficult to discuss certain issues related to clinical nursing education than other areas. Issues about clinical competence assessment were especially difficult to tackle. However, other students said that they did not feel limited or influenced by the nurse educators’ presence during mixed group discussions.

*“I did not find that the presence of the nurse educators restricted my engagement in the discussions. In our group, we all shared and spoke freely.” (P1, FGS)*.

The e-learning designers also emphasized that some nurse educators tended to become dominant during group discussions. They highlighted a tendency among a few nurse educators to promote their own opinions, defending existing practices rather than being responsive to the students’ experiences and contributions. Two of the e-learning designers said:

*“I do not know why this is the case, but some nurse educators became very dominant during the group discussions.” (P1, FGD)*.

*“It was easy to see that the students became insecure when the nurse educators were dominant, then the discussion often died out. Even though there is officially no hierarchy in Norway, a student nurse looks up to a nurse educator. I believe that it is challenging for students to say what they mean when an authority figure is present.” (P2, FGD)*.

These power imbalances led the e-learning designers to reflect on the value of arranging separate workshops with the various stakeholder groups in the ideation phase as well as the problem identification phase. They felt that this would ensure shared decision power and influence. One of the e-learning designers said:

*“When reviewing the ideation phase and production, it would perhaps have been more useful to do it with groups of just students and just nurse educators to avoid the power imbalance.” (P3, FGD)*.

Some student nurses also raised this issue and discussed how power imbalances could be better accommodated. One student said:

*“We students should perhaps have been better aligned as a group before we were mixed with nurse educators and registered nurse mentors. That might have helped us to stand together as a group and stand up more for what we think.” (P1, FGS)*.

The nurse educators did not raise the same concerns about power imbalances. They were more concerned about the pedagogical quality when co-creating digital educational resources. Most of the nurse educators found it interesting and enjoyable to collaborate with the students. However, a few said that during the joint workshop, they had at times found it difficult to take the student nurses’ experiences and opinions seriously. When reflecting on the students’ input during the workshop, one nurse educator commented:

*“We laugh at the students sometimes because of what they say, but we do need to take them seriously. That became clear to me today.” (P6, FGE)*.

### Contextual factors influenced the participants’ overall engagement

The stakeholder groups reflected upon and highlighted several contextual issues that influenced their engagement during the co-creation workshops. These issues included how the workshops were organized and structured and the degree of the participants’ digital competence and attitudes toward digital educational resources for clinical nursing education. Across the stakeholder groups, all participants emphasized the value of how the workshop was pre-organized, including that they received a clear agenda and questions to reflect upon beforehand. They also appreciated that the joint workshop started with lunch, during which all stakeholders were allowed to talk and mingle before participating in the co-creation process.

*“I really appreciated that we started with lunch together, it gave a good framework for the workshop.” (P6, FGE)*.

The duration of the workshop was another issue raised across the stakeholder groups. Some participants were impressed by the efficiency of the workshop and all issues they managed to discuss in the groups. However, the nurse educators and e-learning designers would generally have preferred a full-day workshop rather than a half-day workshop because this would have allowed more thorough discussions and ideations of solutions.

*“I felt that time was a bit short. Several things were sort of rushed through a bit. We didn’t get to go into things in depth. It might have been better as an all-day event.” (P2, FGD)*.

Another e-learning designer suggested that having a facilitator in each group might have been helpful for managing time and progress. The e-learning designers suggested that the research team facilitating the workshops could have been more active in the separate group discussions to ensure inclusivity and progress.

*“In the group I participated in, it would have been nice to have a facilitator or a moderator because the registered nurse mentors said very little.” (P1, FGD)*.

*“We didn’t get much time to discuss media types and possible solutions. Many of the participants were more concerned with discussing general matters about clinical education.” (P2, FGD)*.

Several participants, especially those who participated in groups of more than five participants, stressed that the group sizes were too large, and that this tended to restrict engagement. Both the e-learning designers and students noticed and commented that it was not always easy to contribute, especially for non-native speakers.

*“I tried to interrupt the group conversation to let the nurses with a migrant background contribute, but it was not easy for them to speak up.” (P3, FGD)*.

*“I noticed that one non-native registered nurse mentor did not join in the discussion much, although they sometimes came up with some contributions. It was easier for the Norwegian speakers to get involved.” (P2, FGD)*.

Poor digital literacy skills and attitudes toward digital educational resources for use in clinical nursing education were other issues that were noted to have affected the participants’ engagement, especially during the ideation phase and the joint workshop. The e-learning designers and some students commented that several nurse educators had limited knowledge of digital educational resources and solutions and poor digital literacy skills/digital competence in general, which they felt restricted their input. The e-learning designers noticed that the students were quicker to embrace different media formats and resources, and the nurse educators were more reluctant and concerned, asking probing questions about the use of the different media formats.

*“The students are young, they are media creators. For them, it is completely natural to create media content. For those aged 40–50 and above, it is a foreign language.” (P1, FGD)*.

Varying attitudes among the nurse educators also existed toward the use of digital educational resources in clinical nursing education. Some were more skeptical than others. Both student nurses and e-learning designers commented that the nurse educators’ attitudes toward digital educational resources appeared to influence their engagement in the ideation phase. The students therefore emphasized the value of having the e-learning designers in the workshops because they assisted in and supported discussions about digital solutions in the ideation phase. One student nurse said:

*“The fact that the e-learning designers were involved [in the workshops] made it easier to provide input, discuss digital resources and media formats that we as students wanted to be included in the digital learning resource.” (P7, FGS)*.

## Discussion

The findings of this study suggest that two key aspects of stakeholders’ experiences in participation in co-creation workshops could be targeted to enhance the quality of clinical nursing education in nursing homes by means of co-creation of digital educational resources. The first is the value of co-creation initiatives in clinical nursing education and the second is the barriers and facilitators to stakeholder engagement in co-creation initiatives for clinical nursing education. The discussion is structured around these two key aspects, with other issues discussed as they arise, including practical implications of the study findings.

### Value of co-creation initiatives in clinical nursing education

Our findings suggest that co-creation through workshops is a novel approach that actively engages students and nurse educators and ensures that they contribute as important partners in an ongoing dialogue about the quality of clinical education in nursing homes. Co-creation of novel educational resources with other key stakeholders in nursing education is also an efficient and enjoyable way to share and learn about each other’s experiences. In line with previous reports [26, 27], our findings suggest that participation in co-creation initiatives for nursing education is valuable at the individual and professional levels because it facilitates self-reflection and reflective dialogues. This consequently provides valuable insights into and greater understanding of the needs and concerns of other stakeholders. Our findings also suggest that participation in co-creation workshops may challenge the traditional norms and practices about the roles of academics and students within higher education, paving the way for collaborative approaches and greater democratization of the educational process [13]. The student nurses in our study emphasized that their perspectives were taken seriously and valued through participation in the co-creation workshops. Several nurse educators also acknowledged the importance of taking the students’ perspective into consideration. The e-learning designers highlighted the importance of including end users in designing and developing digital educational resources. These findings are in line with other reports highlighting the value of empowering student nurses [7, 13, 26]. For example, Watson et al. [15] demonstrated that participating in co-creating curriculum content for care home nursing positively changed the attitudes of student nurses about clinical education in aged care. Students are often untapped resources and hold unique perspectives on teaching and learning [13, 28]. Based on our study findings, co-creation initiatives for clinical nursing education are important avenues for fostering greater democratization and collaboration opportunities in educational processes [7, 26]. Including stakeholders (e.g., students) in such activities may encourage and enhance their ability to take an active role in their learning journey [29]. Accordingly, co-creation initiatives may help raise the quality of clinical education, ensuring that key stakeholders are regarded as essential contributors in designing educational resources prepared to meet future challenges and changes, which are occurring at a much faster pace now than ever before [2].

### Barriers and facilitators to stakeholder engagement in co-creation initiatives for clinical nursing education

In this study, there were several barriers to stakeholder engagement in the co-creation workshops but also some issues/strategies that could help improve such engagement. Experience of power imbalances was an important issue noted herein. Even though workshops have been less frequently applied in nursing education than other activities, they are widely used in co-creation approaches in healthcare settings and are considered important catalysts of participant commitment and equalizers of power relations (e.g., [30]). However, power relations appeared to be a challenge in the workshops in this study, indicating the need for such to be reshaped in the future [31]. Our findings suggest that power imbalances might challenge genuine co-creation partnerships between students and nurse educators. Co-creation in higher education therefore does not necessarily automatically have the level of equality required for partnership, which could be a barrier to mutual learning and innovation [13]. This issue is a major concern in co-creation because feeling psychologically secure and experiencing relational equality are essential factors for successful engagement and therefore innovation [32, 33]. In line with other reports [13, 29], we suggest that recognizing and managing power differentials are essential for successful co-creation in clinical nursing education and that a conscious mindset of collaboration and mutuality rather than hierarchical thinking should be developed. A recent literature review [29] stresses that successful co-creation requires educators to be invested in these initiatives, have a positive attitude, and let go of rigid agendas and a sense of control. This entails nurse educators to be willing to consider and apply their clinical and pedagogical expertise, experiences, skills, and knowledge as a mechanism for supporting students’ development and growth through co-creation initiatives in a flexible manner [29] and for potentially improving their own practice [1].

Our findings suggest practical strategies that could help mitigate hierarchy and power imbalances contextually considered a crucial aspect in providing space for students’ voice and agency [29]. First, the participants emphasized that the ratio of nurse educators to student nurses and the group size were important aspects to consider during the joint workshop to ensure inclusiveness and facilitate fruitful conversations and discussions. Second, the combination of separate and joint workshops was considered beneficial, especially by the student nurses and e-learning designers. They felt that this approach safeguarded honest and open reflections to a higher degree than did the joint workshop. This finding resonates with previous data emphasizing the importance of the creation of psychologically safe spaces during and throughout co-creation processes [29], enabling an open exchange of thoughts and ideas among, for example, students and nurse educators. However, this approach relies mainly on nurse educators being able to honor/value students’ unique experiences to empower them by listening openly and actively and promoting a genuine dialogue [29]. Finally, having a formal facilitator to lead the group discussions was considered essential, a structure stressed by Ha and Pepin [27]. The authors suggest that this structure is needed to guide the collaboration and dialogue, especially when co-creation is not a familiar process for either students or educators [27]. Such approach will better ensure that all participants have equal opportunities to participate in the discussions. Hasan and Rahman [34] suggested that higher educational institutions should conduct classes and provide syllabi and workshops to train and encourage students and staff to be more participative in co-creation processes. In our study, the lack of digital literacy skills, poor attitudes toward digital educational resources, and lack of pedagogical competence among the stakeholders participating in the co-creation workshops were all perceived as potential barriers to successful engagement. This finding is consistent with concerns raised in previous studies [7, 13, 35], indicating the need for a conscious approach to co-creation when it is appropriate to be used based on clear goals and competence needs [1]. Nevertheless, we also found that the participants shared their experiences and knowledge during the workshops, which contributed to mutual learning associated with increased pedagogical insights and digital literacy skills. This finding is supported by the claims of Könings et al. [36] that active engagement of learners in educational design can support change and professional development of educators and learners alike. However, the lack of digital competence underlines an important implication supporting the value of having e-learning designers actively involved in co-creation workshops when developing digital educational resources for nursing education. Pedagogically, e-learning designers might represent a more neutral group of stakeholders, which may help balance inherent power structures. These designers are considered to possess essential expertise and competence that can help translate opportunities and ideate digital solutions that are both functional and useful for clinical nursing education. The involvement of e-learning designers can therefore help ensure inclusiveness and successful engagement of all stakeholders in co-creation processes, unlocking the full potential of digital learning resources.

Despite the present findings, we recommend continuous evaluation of co-creation initiatives within nursing education to better account for, document, and learn from the factors and barriers to success supported by other reports [7, 37, 38]. A recent literature review on co-constructing knowledge in higher education [29] emphasizes caution to a one-size-fits-all approach in co-creation initiatives. The author [29] calls for attention toward employment of a variety of co-creation activities to better account for individual, diverse, and marginalized student needs. Even though participation in the co-creation workshops was experienced as fun and enjoyable in our study, we acknowledge the challenge concerning inclusiveness in this co-creation initiative, which may have been a potential barrier to ensuring diversity during study enrollment and participation [1, 36].

### Limitations

This study has some limitations. It was based on a small sample from a single educational institution, which restricts the transferability of the findings. However, Bovill [39] recommended starting on a small scale when co-creating in a new field such as clinical nursing education. The novelty of the study and its findings are also likely to be relevant in both national and international contexts. However, the study is prone to potential research bias. The data were collected by researchers with a background in nursing, entailing prior contextual understanding. Moreover, the researchers participating in and facilitating the co-creation workshops and interviews were nurse educators at the same educational institutions as the participants, creating a dynamic of insider research [40]. This approach may have inhibited the participants to speak less freely than they would have if an external research team was leading the co-creation initiatives and interviews owing to power imbalances and researcher ambitions. Conversely, previous research suggests that familiarity and established intimacy may promote more efficient knowledge-sharing [40]; thus, the use of the insider team might also have strengthened the study. To avoid research bias, we applied triangulation during the data analysis, in which three of the authors were not involved in the interviews. These three authors actively participated and reflected upon the findings, as did authors from other educational institutions who were not involved in the active co-creation process (e.g., [41]).

## Conclusions

This study shows that co-creation through workshops is a novel, enjoyable, and instructive approach that effectively facilitates knowledge exchange and highlights the experiences and needs of stakeholders, especially student nurses. However, the co-creation of digital educational resources through workshops for nursing education is not without challenges. Co-creation initiatives to enhance the quality of clinical nursing education have potential that merits further development and consideration in education and research. The relative novelty of co-creation in clinical nursing education implies that there are still knowledge gaps to be explored. Further research must address strategies on how to optimally involve and engage student nurses in co-creation initiatives to improve learning and facilitate achievement. The influence of co-created educational resources and stakeholders’ motivation, barriers, and facilitators to participation must also be evaluated. Finally, further research on co-creation initiatives that promote more comprehensive and inclusive approaches and perspectives is needed to ultimately enhance the quality and effectiveness of clinical nursing education.

### Supplementary Information

**Additional file 1. **Interview guide.

## Data Availability

The dataset used and analyzed during the current study is available from the corresponding author on reasonable request.

## Abbreviations

RN: Registered nurse
FGS: Focus group students
FGE: Focus group educators
FGD: Focus group e-learning designers
