# Supplementary material for: Exploring stakeholders’ experiences in co-creation initiatives for clinical nursing education: a qualitative study
Source: BMC Nurs. 2023 Nov 6;22:416. doi: 10.1186/s12912-023-01582-5 (PMC10626696; doi:10.1186/s12912-023-01582-5)
Supplement: Supplementary file 1 — Additional file 1. Interview guide. [file 12912_2023_1582_MOESM1_ESM.docx]

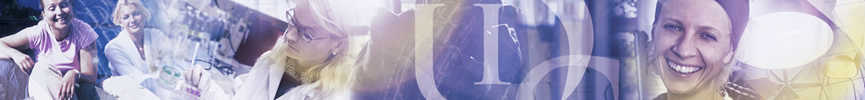


**CO-creative workshops interview guide all stakeholders**

The focus is to explore the participants' experiences related to participation in co-creative workshops (Process oriented) and the utility they think the digital educational resource will have (product oriented)

**Questions**


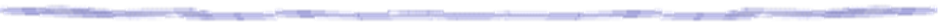


1. How has it been to participate in the workshops – can everyone describe their experiences (Process oriented)

Follow-up questions:

1. Something that was fun, educational, difficult, challenging – describe and give/provide an example(s)
2. Has anything changed your perceptions/thoughts/needs because of participating in the workshops?
3. After participating in co-creative workshops are there things/ideas/thoughts you want to take with you from this experience – describe
4. What do you think about the utility value of the digital educational program that we are co-creating? (Product oriented)
5. What are you experiences related to the structure and organization of the co-creative workshops (Process oriented) (potential follow up questions what your experiences are related to time, number of participants, group composition, facilitation issues, improvements)
